# The Analgesic and Anti-inflammatory Effects of Partially Purified Polysaccharide Fractions of Cell-free Medium and Biomass of *Spirulina platensis* PCST5

**DOI:** 10.5812/ijpr-136661

**Published:** 2023-07-19

**Authors:** Mohammad Dehghanizadeh Baghdadabad, Nima Naderi, Vahideh Atabaki, Mohammad Ali Faramarzi, Maryam Tabarzad, Tahereh Hosseinabadi

**Affiliations:** 1 Department of Pharmacognosy, School of Pharmacy, Shahid Beheshti University of Medical Sciences, Tehran, Iran; 2 Department of Pharmacology and Toxicology, School of Pharmacy, Shahid Beheshti University of Medical Sciences, Tehran, Iran; 3 Department of Pharmacognosy and Pharmaceutical Biotechnology, Faculty of Pharmacy, Hormozgan University of Medical Sciences, Bandar Abbas, Iran; 4 Department of Pharmaceutical Biotechnology, Faculty of Pharmacy, Tehran University of Medical Sciences, Tehran, Iran; 5 Protein Technology Research Center, Shahid Beheshti University of Medical Sciences, Tehran, Iran

**Keywords:** Cyanobacteria, Pain, Inflammation, Formaldehyde, Carrageenan, Polysaccharide

## Abstract

**Background:**

*Spirulina* is a cyanobacteria species containing various bioactive compounds. *Spirulina* is a known source of nutrients in some traditional diets. Different activities have been reported for various extracts of *S. platensis*.

**Objectives:**

In this study, the polysaccharide content of culture media and biomass extract of one species of *Spirulina* was partially purified, and its analgesic and anti-inflammatory effects were evaluated.

**Methods:**

*Spirulina platensis *PCST5 was cultured in a sterile Zarouk medium at 27°C and 16/8h of light/ dark exposure cycle for 25 days. Then, the polysaccharide content of biomass and cell-free culture medium samples (BPSs and CFPSs, respectively) was partially purified. The analgesic and anti-inflammatory effects were evaluated using animal models.

**Results:**

*16S rRNA* gene analysis confirmed that the organism was genetically similar to *Spirulina platensis*. The CFPSs (30 and 100 mg/kg) and BPSs (30 mg/kg) significantly reduced pain-related behaviors in rats. Similarly, all samples could significantly reduce carrageenan-induced paw inflammation volume compared with the control group. Our results suggest *Spirulina*'s polysaccharide fractions (CFPSs and BPSs) had significant analgesic and anti-inflammatory effects.

**Conclusions:**

Since *Spirulina* is a readily available source of bioactive compounds, finding such potent anti-inflammatory and anti-nociceptive compounds can provide promising leads for novel drug development.

## 1. Background

The genus *Spirulina* belongs to the Oscillatoriaceae family and contains a group of filamentous cyanobacteria having the helical shape of trichrome, known as a unique morphological characteristic of this genus (1, 2). *Spirulina* or *Arthrospira* is an appreciated source of nutrients in the traditional diet of some countries. It contains high amounts of proteins (60 - 70%), polysaccharides (6 - 12%), various fatty acids (linoleic and gamma-linolenic acids), amino acids, minerals, and vitamins, especially B-complex and pigments. Spirulina's simple commercial production process and its nutritional importance have made this organism a valuable food supplement, animal feed additive, and drug development ingredient (1, 3, 4).

The anti-inflammatory characteristics of *Spirulina* sp. extracts have been well-documented. Chen et al. showed that *Spirulina* sp. could reduce the mRNAs levels of inflammation-related proteins in microglial cells (BV-2 cell line), including inducible nitric oxide synthase (iNOS), cyclooxygenase-2 (COX-2), tumor necrosis factor-alpha (TNF-α), and interleukin 6 (IL-6). Therefore, this microorganism can be a therapeutic candidate for microglia-associated neuronal damage (5). *Spirulina* sp., as a complementary drug, exhibited an ameliorative role in aspirin-induced gastric ulcers by alleviating oxidative stress and inflammation. This effect is demonstrated to be accompanied by reducing the level of inflammatory mediators (6). It is demonstrated that *Spirulina* sp. can control acute ear inflammation induced by 12-O-tetradecanoylphorbol-13-acetate (TPA) in mice (7).

Recently a wide variety of biological activities have been reported from polysaccharides (PSs) extracted from *Spirulina* sp., including anti-ulcer, anti-oxidant, anti-inflammatory, immunomodulatory, anti-viral, and anti-coagulant activities, as well as, the inhibitory activity on corneal neovascularization (8, 9). Several in vitro studies reported that PSs of *Spirulina* sp. could increase cell nucleus enzyme activity and DNA repair synthesis (10).

## 2. Objectives

In the present study, we first extracted PS content from *Spirulina platensis *PCST5 biomass and the excretion media. We then evaluated their effects on inflammation and pain-related behavior by carrageenan and formalin in vivo tests, respectively.

## 3. Methods

### 3.1. Chemicals

Zarouk Culture medium was prepared as described by De Oliveira et al. with the following components, KNO_3_, MgSO_4_.7H_2_O, CaCl_2_.2H_2_O, K_2_HPO_4_, Na_2_CO_3_, NaCl, H_3_BO_3_, MnCl_4_.H_2_O, ZnSO_4_.7H_2_O, Na_2_MoO_4_.2H_2_O, CuSO_4_.5H2O, Co(NO_3_).6H_2_O, and EDTA, FeSO_4_.7H_2_O, K_2_SO_4_, and NaHCO_3_ (All from Merck Chemicals, Germany) (11).

### 3.2. Preparation and Cultivation of Spirulina sp.

*Spirulina platensis *PCST5 sample was donated by the Department of Biotechnology, School of Pharmacy, Tehran University of Medical Sciences, Tehran, Iran, and was cultivated in a flask containing Zarouk culture medium (200 mL) at 27°C in germinator (Grouc, Iran) under 16/8 h light/dark cycle, and 70% humidified condition, then passaged after 21 - 25 days.

### 3.3. 16S rRNA Amplification and Sequencing

One mL of cyanobacterium culture was collected, and the biomass was separated by centrifugation at 10000 ×g for 15 min. DNA was extracted using a genomic DNA extraction kit (GeneJET, Thermofisher). A segment of *16S rRNA* gene regions were amplified by PCR using the 16S-27F (5’-AGAGTTTGATCCTGGCTCAG-3’) and 16S-1492R (5’-GGTTACCTTACGACTT-3’) primers (12).

The amplification process was 34 cycles of amplification starting with 3 min at 94°C, then followed by repeated cycles of 1 min at 94°C, 1 min at 55°C, and 1 min at 72°C, using Taq DNA polymerase. The termination cycle was 10 min at 72°C. The PCR products were analyzed on 1.5% (w/v) agarose gel using tris-borate-EDTA (TBE) buffer. The PCR product was also sequenced. The national center for biotechnology information (NCBI) nucleotide BLAST tool was used to find homologous and other close sequences.

### 3.4. Extraction of PSs

#### 3.4.1. Extraction of PSs from Cell Free Culture Medium (CFPSs)

The complete culture medium (200 mL) was transferred into several 15 mL Falcone tubes and centrifuged at 10000 ×g for 30 min, and the supernatant was collected and condensed for 12 h under nitrogen gas. Absolute ethanol (EtOH 99.5%) was added, and the samples were stored in an -80°C freezer (Jal Tajhiz, Iran). After 24 h, the samples were centrifuged (10000 ×g, 20 min), then the obtained pellets were collected and washed with cool acetone, then dried at room temperature for 24 h. In the next step, the dried pellet dissolved in distilled water (300 mL) to obtain a clear solution. The solution was dialyzed against distilled water for 72 h using a 12 kDa dialysis bag (SIGMA D-9277, Germany). At the end of the dialysis process, the final samples were transferred to Falcone tubes and lyophilized (Freeze-Dryer, Christ, Germany) (13).

#### 3.4.2. Extraction of PSs from Biomass (BPSs)

Biomass was separated from the culture medium and dried by lyophilization. Ten grams of dried biomass was suspended in distilled water, then NaOH (1 M) was added to the solution to set its pH value at 10.25. This solution was preserved at 89.24°C for ten h and then centrifuged (4300 ×g, 20 min). Subsequently, polysaccharide content was precipitated by five volumes of EtOH at -20°C and then re-centrifuged (4300 ×g, 10 min). The pellet was suspended in distilled water (pH 7.0) and, after adding papain 3% (Sigma, Germany), incubated at 50°C for 2.5 h. Then, the enzyme was inactivated by boiling, and 5 % trichloroacetic acid was added and kept at 4°C overnight. Then, H_2_O_2_ in a final concentration of 5% was added (kept at pH 8.0, 55°C, 2h). Again, 5 volumes of EtOH (95%) were added (4°C, 24 h), and then, the precipitate was separated from the solution by centrifugation (4300 ×g, 10 min), washed in acetone, and dried (3).

#### 3.4.3. Bradford Assay

Bradford analysis was performed according to the standard method (14) using TaKaRa Bradford Protein Assay Kit (T9310, Japan). First, a serial dilution (0 - 1000 µg/mL) of bovine serum albumin (BSA) standard samples was prepared. Then, 20 µL of standard and test samples were added to 1000 µL of Bradford reagent in separated microtubes and incubated at room temperature for 5 min. Then, the absorbance of mixtures was recorded at 595 nm (Thermo Scientific™ NanoDrop™ One, USA).

### 3.5. In Vivo Experiments

#### 3.5.1. Animal Groups

Male Wistar rats (120 - 150 g for the formalin test, 280 - 320 g for the Carrageenan test) were purchased from Pasteur Institute (Tehran, Iran). The rats were held in special cages in a 12/12 h light/dark cycle in controlled conditions at 25 ± 2°C and 30 ± 10% humidity, with free access to standard food and water. This study was performed according to the instructions provided by the local ethics committee for animal experimentation. We endeavored to minimize the pain or discomfort of animals in all experiments. Six and seven rats in each group were studied in formalin and carrageenan tests, respectively. Animal studies were conducted concerning ethical considerations and animal rights protocols. The research proposal was investigated and approved by the Ethical Committee of Shahid Beheshti University of Medical Sciences, Tran, Iran (IR.SBMU.PHARMACY.REC.1399.368)

#### 3.5.2. Preparation of Drugs

The CFPSs (30 and 100 mg/mL) and PSs (30 mg/mL) were prepared in normal saline, and ibuprofen as the positive standard (50 mg/mL) was prepared in dimethyl sulfoxide (Merck, Germany). 5% formalin and 2% carrageenan solutions (Merck, Germany) were prepared in normal saline. Normal saline (GHAZI, Iran) was the negative control (1 mL/kg).

#### 3.5.3. Carrageenan-Induced Paw Edema Test

The CFPSs (30 and 100 mg/kg), BPSs (30 mg/kg), ibuprofen (50 mg/kg), and normal saline were injected intraperitoneally. After 30 minutes, 0.1 mL of 2% w/v carrageenan solution (in saline) was administered into the sub-plantar tissues of the rats’ left hind paws to trigger inflammation. Before carrageenan injection, the rat paw volume was measured by immersing it in a mercury column placed on an electronic balance. The weighting process was repeated 3 and 4 h after the carrageenan injection. The difference between rat paw volumes before and after carrageenan injection was calculated by the formula ρ =m/v, in which m stands for weight, v for volume, and ρ for mercury density (13.534 g/cm^3^) (15).

#### 3.5.4. Formalin Test

The study was performed after intraperitoneal administration of CFPSs (30 and 100 mg/kg), BPSs (30 mg/kg), ibuprofen (50 mg/kg), and normal saline. After 30 min, formalin 5% (0.04 mL) was injected into the dorsal surface of the rat's hind paw to induce a pain response. The pain-related behavior of the rats was evaluated in 15s intervals for a total of 60 min and scored based on the method of Dubuisson and Dennis, in which the 0 score is equal to normal weight bearing on the injected paw, the 1 score means limping during locomotion or resting the paw lightly on the floor, the 2 score means elevation of the injected paw so that at most the nails touch the floor. The 3 score is licking, biting, or shaking the injected paw (16).

#### 3.5.5. Statistical Analysis

The data were calculated as mean ± SEM and statistically analyzed using Prism 6 (Graphpad Software Inc.). The changes in pain-induced behavior among the treatment groups at different times were assessed by two-way analysis of variance (ANOVA) followed by Tukey post-test. One-way ANOVA with post-hoc Dunnett’s test was used to compare the area under the curve (AUC) of pain score. The data of the carrageenan test were also analyzed by two-way ANOVA with Dunnett’s post-test. P < 0.05 was considered significant.

## 4. Results

### 4.1. Molecular Confirmation of Spirulina platensis PCST5

**Figure 1.**
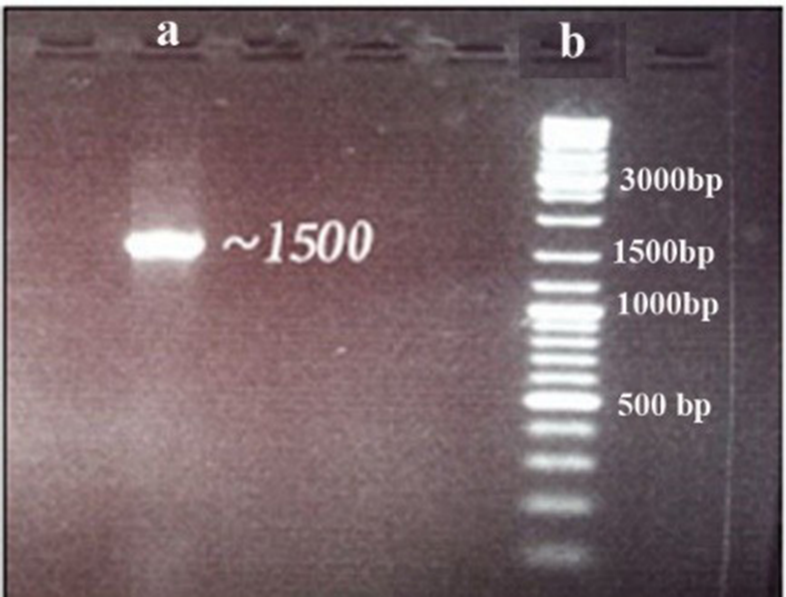
*16S rRNA* amplification and sequencing, (A) PCR amplification product, (B) DNA ladder (GeneRuler SM 033)

PCR amplification of the *16S rRNA* gene resulted in a single sharp band with about 700 bp (Figure 1). The sequence of the amplified *16S rRNA* gene segment (1286 bp) showed 97% similarity with *Spirulina platensis* after blasting using the NCBI BLAST tool (E-values ≤ 10^-20^).

### 4.2. Extraction of PSs from Spirulina Cell-free Culture Medium and Biomass

The cell-free culture medium was separated from biomass, dried (8 g), and dissolved again in distilled water. This solution is dialyzed against distilled water (12 kDa). Finally, large macromolecules weighted higher than 12 kDa were extracted, and most of their components were expected to be PSs or proteins secreted in the culture medium. The final solution was freeze-dried (450 mg). The amount of protein in cell-free culture was determined using Bradford assay, which was less than 1% of dried extract.

### 4.3. Anti-inflammatory Effect of the Spirulina Extracts on Carrageenan-induced Inflammation

**Figure 2.**
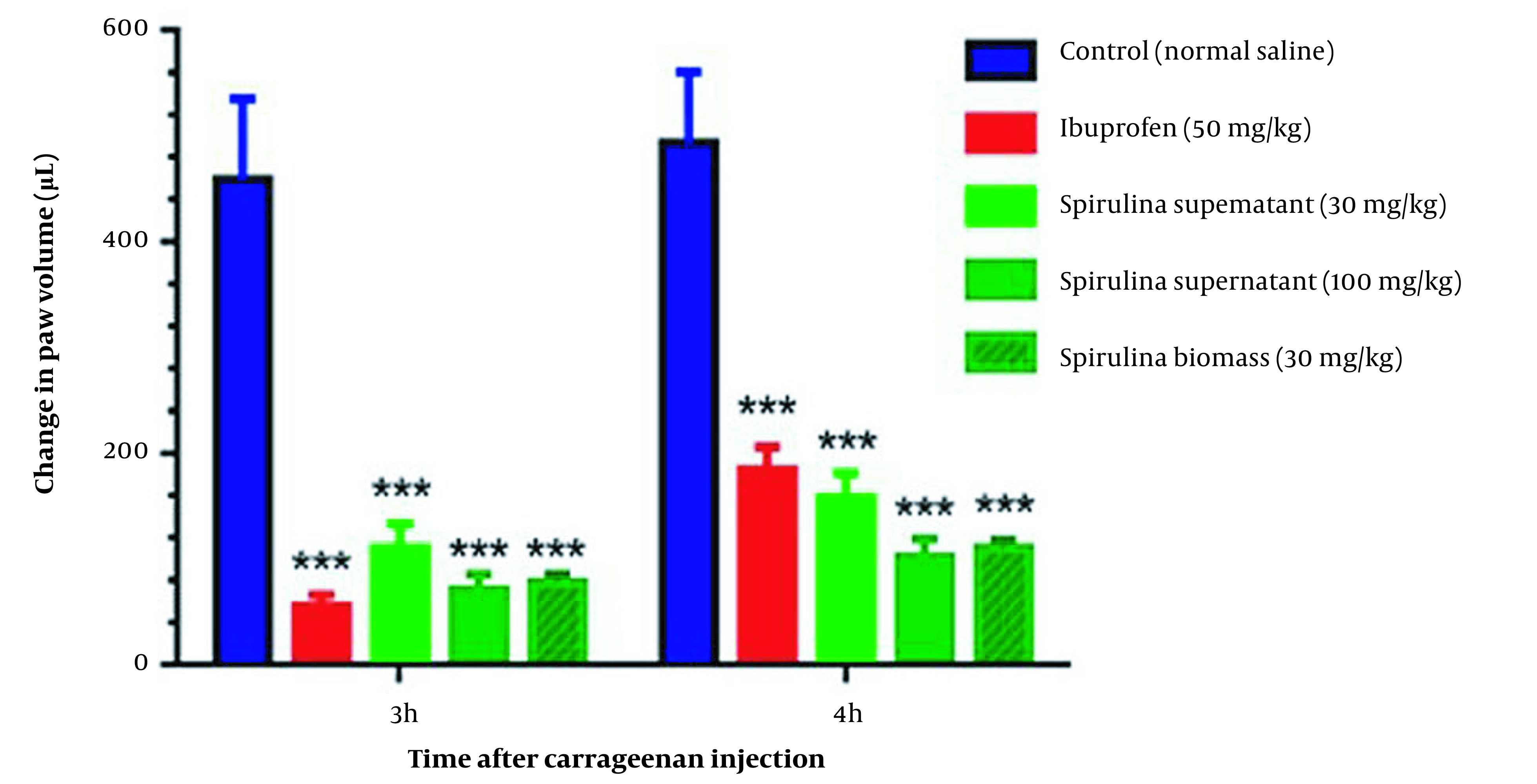
Comparison of the anti-inflammatory effect of polysaccharides extracted from *Spirulina platensis *PCST5 in different doses on carrageenan-induced paw edema. The data indicate the difference in paw volume at 3 and 4 h after carrageenan injection. The results are shown as mean ± SEM (n = 7). *** P < 0.001 significant difference compared to the control group (normal saline). *Spirulina platensis *PCST5 supernatant indicates the extracts from a cell-free culture medium. The differences between groups were not significant.

Carrageenan test confirmed that the 30 and 100 mg/kg of CFPSs and the dose of 30 mg/kg of BPSs, exhibited good anti-inflammatory activity. Statistical analysis showed significant inflammation reduction in treatment groups (P < 0.001) compared to control groups, without significant interaction between time and dose of samples (P = 0.5972) in the carrageenan test. In addition, CFPS and BPS extracts could significantly reduce carrageenan-induced paw edema compared to the control group in both times of 3 and 4 h (P < 0.001) (Figure 2). The amount of volume reduction in the site of inflammation after 3 h was about 78 ± 2.2%, 82 ± 1.8%, 82 ± 2%, and 89 ± 2.1% for CFPSs 30 mg/kg, CFPSs 100 mg/kg, BPSs 30 mg/kg and ibuprofen 50 mg/kg, respectively. The inflammation volume reduction after 4h was calculated at about 59 ± 1.3%, 73 ± 2%, 67 ± 1.8%, and 56 ± 1.9% for CFPSs 30 mg/kg, CFPSs 100 mg/kg, BPSs 30 mg/kg and ibuprofen 50 mg/kg, respectively. The effect of *Spirulina* extracts on reducing the inflammation volume after 4 h was greater than ibuprofen 50 mg/kg.

In the carrageenan test, 100 mg/kg dose of the CFPSs extract and 30 mg/kg of the BPSs extract showed the strongest anti-inflammatory effects. These effects of the CFPSs and BPSs extracts were similar to the activity of ibuprofen at 50 mg/kg.

### 4.4. The Anti-nociceptive Effect of Spirulina Extracts on Pain-related Behavior

**Figure 3.**
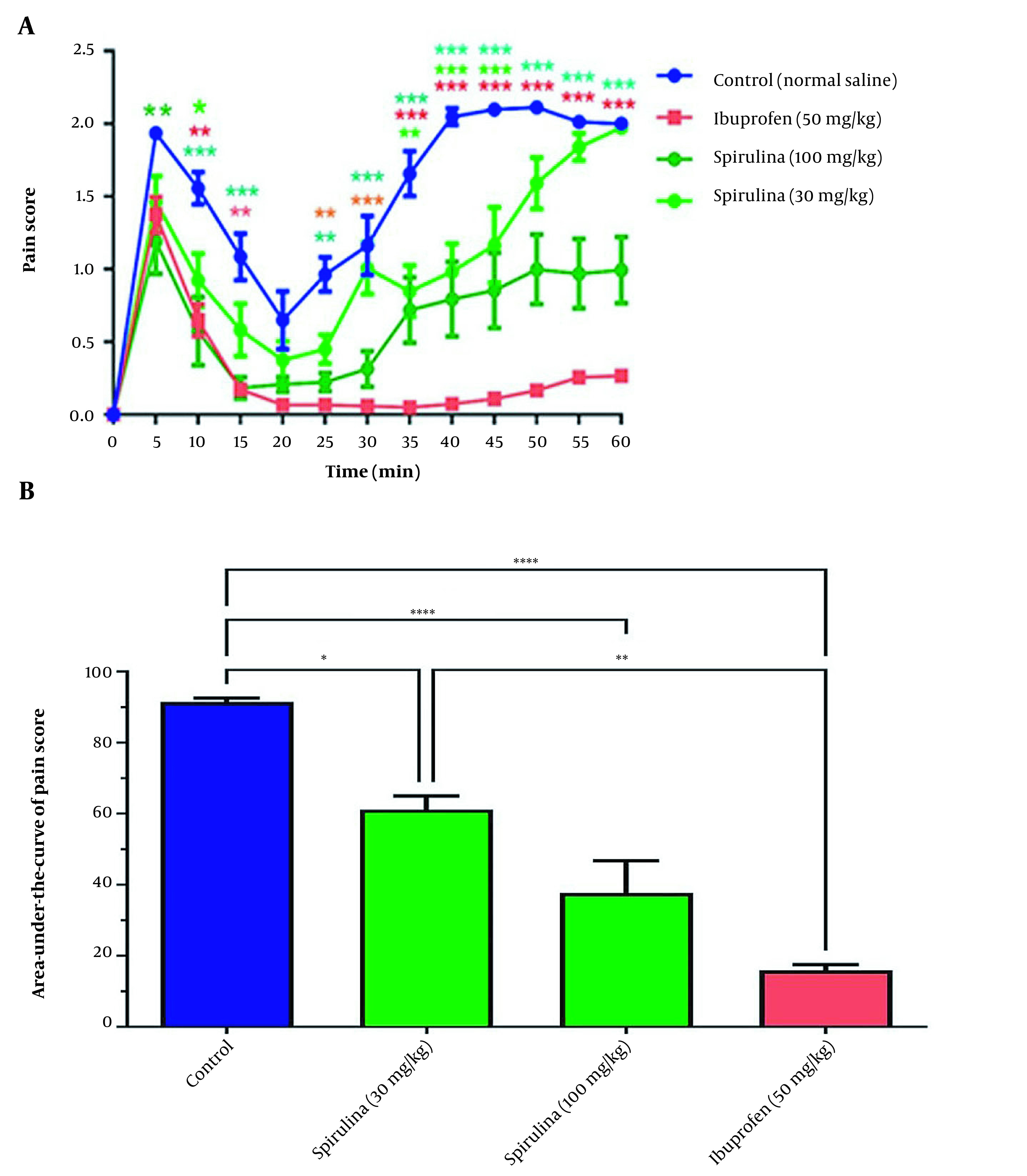
The effect of administration of CFPSs extracted from *Spirulina platensis *PCST5 on pain-related behavior in the formalin test. (A) Overall changes in pain score observed during 60 minutes. (B) The AUC of pain score depicted in Figure A, calculated separately for each rat. All drugs, as well as control, were administered intraperitoneally 30 minutes before the start of the test. The control group received normal saline in the same volumes. The results are shown as mean ± SEM (n = 6). * P < 0.05, ** P < 0.01, *** P < 0.001 and **** P < 0.0001.

Statistical analysis showed that the effect of treatment on pain score in rats treated with the extract of cell-free medium was significant over time (P < 0.0001). The rats treated with 30 mg/kg of the CFPSs had significantly decreased pain scores at 10, 35, 40, and 45 min, compared to the control group. This effect was also observed in the groups treated with 100 mg/kg of the CFPSs extract, 5, 10, 15, and 25 - 60 min. Similarly, a decrease in pain score at 10, 15, 25 - 60 min was observed in the groups treated with 50 mg/kg standard ibuprofen (Figure 3A). As shown in Figure 3B, administration of the CFPSs extract (both doses) significantly changed the pain score curve and its area under the curve (AUC) as an indicator of total pain relief (P < 0.0001). Further analysis demonstrated a significant decrease in the AUC of the pain score curve in rats treated with the CFPSs extract (30 and 100 mg/kg) and ibuprofen (50 mg/kg) as positive standard compared to the control group. The differences between the effect of *Spirulina* 30 mg/kg and *Spirulina* 100 mg/kg on pain were not significant.

**Figure 4.**
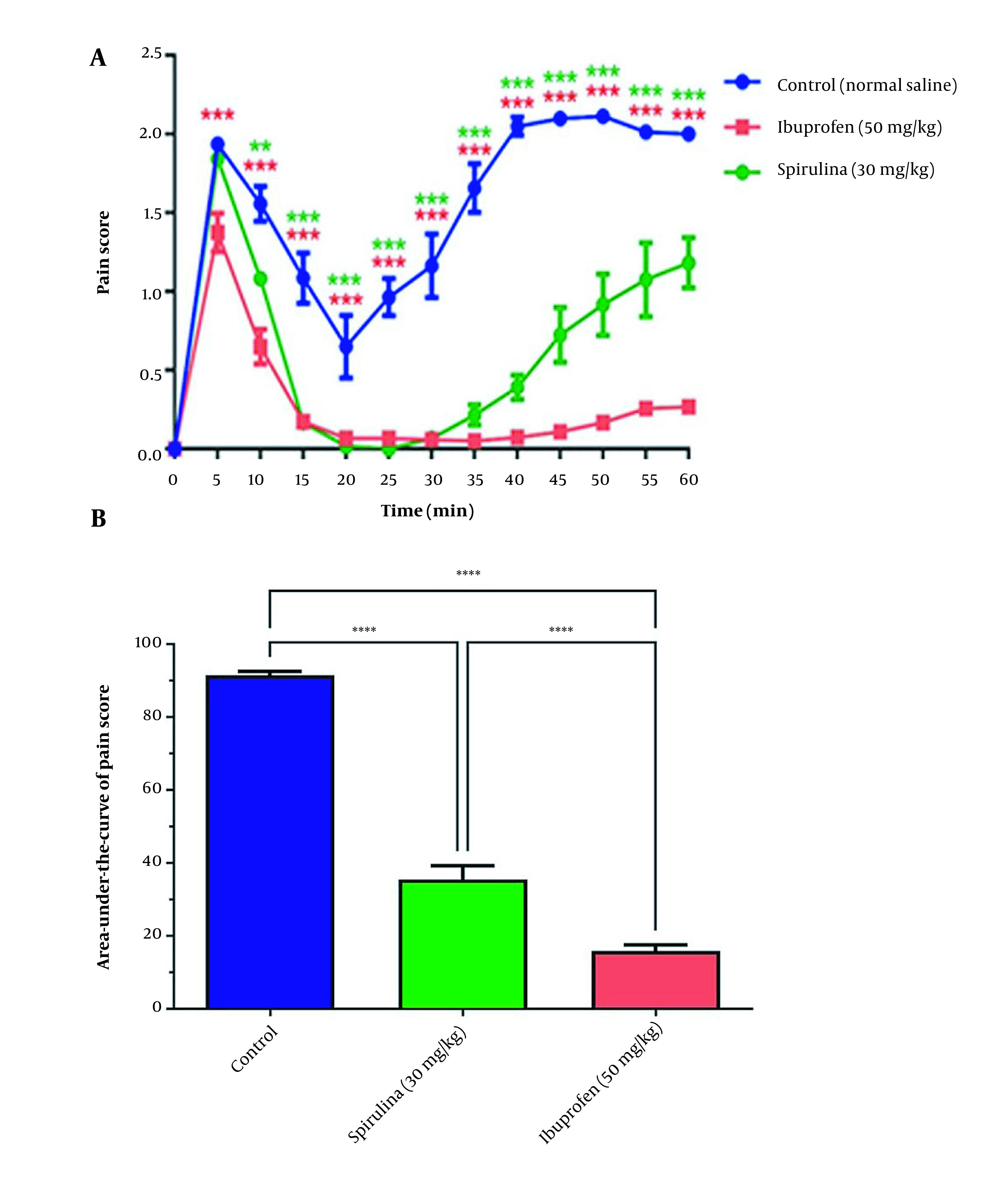
The effect of administration of *Spirulina platensis *PCST5 BPSs extracts 30 mg/kg on pain-related behaviors in the formalin test. (A) Overall changes in pain score observed during 60 minutes. (B) The AUC of pain score depicted in Figure A, calculated separately for each rat. All drugs, as well as controls, were administered intraperitoneally 30 minutes before the start of the test. The control group received normal saline in the same volume. The results are shown as mean ± SEM (n = 6). *** P < 0.001 and **** P < 0.0001.

In addition, treatment with 30 mg/kg of the BPSs extracts significantly reduced pain scores in 10 to 60 min, compared with the control group (Figure 4A). The administration of the BPSs extract showed remarkable changes in the AUC of the pain score curve compared to other extract treatment groups (P < 0.0001). In addition, a significant reduction in the AUC of pain score in rats treated with the BPSs extract (30 mg/kg) concerning the control group (P < 0.001) was confirmed (Figure 4B).

## 5. Discussion

Previously, analgesic, anti-inflammatory, and sedative activities of the extracts of several cyanobacteria such as *Oscillatoria annae* (17), *Oscillatoria willei* (18), *Fischerella* sp. (19), and *Dunaliella salina* (20), have been reported. Besides, cyanobacterial toxins such as saxitoxins have shown analgesic or muscle anesthesia activities (21). *Spirulina* sp. is known as a cyanobacterium species with a wide range of valuable biological activities. A cyanobacterial protein, phycocyanin, is a well-recognized bioactive molecule in drug research (22).

According to the results, it can be suggested that the analgesic (anti-nociceptive) effect of the *S. platensis *PCST5 extract, mainly of PSs and small peptides, may be related to inhibiting inflammatory pathways. It was previously reported that the aqueous extract of a marine cyanobacterium, *Trichodesmium erythraeum*, showed anti-inflammatory activity in carrageenan-induced inflammation in rats at a high dosage (500 mg/kg). This extract could inhibit the inflammation volume by 57.5 ± 5.5 % (23). Our study observed 78% and 82% reduction in inflammation volume by CFPSs 30 mg/kg and both CFPSs 100 mg/kg and BPSs 30 mg/kg, respectively. Therefore, *S. platensis *PCST5 PS extract is a more promising natural anti-inflammatory compound. However, no significant difference between these groups was observed. Moreover, this extract had roughly similar anti-inflammatory effects to ibuprofen 50 mg/kg. It has also been demonstrated that the mega molecular polysaccharide sacran extracted from cyanobacterium *Aphanothece sacrum* has topical anti-inflammatory effects at concentrations of 0.01 and 0.05% (w/v) in the ultimate product (24). Moreover, Malyngamide F, a lipo-peptide of a filamentous cyanobacterium *Lyngbya majuscule*, showed anti-inflammatory and anti-nociceptive effects in animal models through reducing the levels of inflammatory cytokines, PGE-2, IL-6, and TNF-α (25).

Regarding the results of our study, PSs extracted from *S. platensis *PCST5 cell-free culture medium (CFPSs) at doses of 30 and 100 mg/kg and *S. platensis *PCST5 BPSs extract at the dose of 30 mg/kg could significantly reduce the AUC of pain scores and could be considered a promising anti-nociceptive agent. The BPSs extract (30 mg/kg) also showed similar activity to those of the CFPSs extract at 100 mg/kg. Increasing the dose of CFPSs extract could improve the pain-relief activity; however, this reduction was not significant. These extracts were more effective than 50 mg/kg ibuprofen for pain relief.

Neekhra et al. studied the dose-related anti-nociceptive activity of *S. platensis* aqueous extract in mice. They found a dose-responsive effect of up to 400 mg/kg, which had comparable activity to pentazocine 10 mg/kg. The anti-nociceptive agents that primarily affect the central nervous system equally inhibit both early and late phases of pain. However, those that affect peripheral nervous systems inhibit the late phase of pain. Late-phase pain inhibition can also result from the effects of the therapeutic agents on inflammatory chemokine and cytokines. Agents that suppressed both phases of pain, such as the *S. platensis* extract, affected both central and peripheral nervous systems (26). The results of our study also confirmed the early and late phase inhibition of pain for CFPSs 100 mg/kg and BPSs 30 mg/kg. However, regarding the CFPSs 30 mg/kg, the pain score increased with time to reach the same level of control.

Recently, Santos et al. reported the anti-nociceptive properties of *S. platensis* biomass and characterized the mechanisms of action that probably were mediated by the opioid system (27). In another study, unnarmicin D, a cyanobacterium Trichodesmium thiebautii peptide metabolite, showed strong binding potential to the μ opioid receptor. Consequently, its derivatives were introduced as promising candidates for treating pain and inflammation, particularly neuroinflammation-related diseases (28).

The effect of 30 mg/kg of BPSs was generally comparable to 100 mg/kg of CFPSs. This may confirm the presence of more potent compounds in the biomass extract than excreted metabolites in a cell-free culture medium. However, more studies need to identify the main bioactive compounds of extracts and their exact mechanisms of action in reducing pain and alleviating inflammation.

### 5.1. Conclusions

In this study, the polysaccharide fractions extracted from the cell-free culture medium (CFPSs) and the biomass (BPSs) of *S. platensis *PCST5 exhibited significant analgesic and anti-inflammatory effects. Based on the results, it can be suggested that the aquatic extract of *S. platensis *PCST5, mainly containing PSs, is a promising candidate for analgesic and anti-inflammatory activities. However, more structural and mechanistic studies are required to identify the main bioactive components and their precise mechanism of analgesic and anti-inflammation actions.

## Data Availability

The extra data, rather than those presented in the study, is available on request from the corresponding author during submission or after publication.
